# Crystallization Behavior of Isotactic Propene-Octene Random Copolymers

**DOI:** 10.3390/polym14194032

**Published:** 2022-09-26

**Authors:** Miriam Scoti, Fabio De Stefano, Angelo Giordano, Giovanni Talarico, Claudio De Rosa

**Affiliations:** Dipartimento di Scienze Chimiche, Università Degli Studi di Napoli Federico II, Complesso Monte S. Angelo, Via Cintia, 80126 Napoli, Italy

**Keywords:** isotactic polypropylene, copolymers, metallocene catalysts, role of defects excluded from crystals

## Abstract

The crystallization behavior of random propene-octene isotactic copolymers (iPPC8) prepared with a homogeneous metallocene catalyst has been studied. Samples of iPPC8 with low octene content up to about 7 mol% were isothermally crystallized from the melt at various crystallization temperatures. The samples crystallize in mixtures of the α and γ forms of isotactic polypropylene (iPP). The relative amount of γ form increases with increasing crystallization temperature, and a maximum amount of γ form (*f*_γ_(max)) is achieved for each sample. The crystallization behavior of iPPC8 copolymers is compared with the crystallization from the melt of propene–ethylene, propene–butene, propene–pentene, and propene–hexene copolymers. The results show that the behavior of iPPC8 copolymers is completely different from those described in the literature for the other copolymers of iPP. In fact, the maximum amount of γ form achieved in samples of different copolymers of iPP generally increases with increasing comonomer content, while in iPPC8 copolymers the maximum amount of γ form decreases with increasing octene content. The different behaviors are discussed based on the inclusion of co-monomeric units in the crystals of α and γ forms of iPP or their exclusion from the crystals. In iPPC8 copolymers, octene units are excluded from the crystals giving only the interruption effect that shortens the length of regular propene sequences, inducing crystallization of the γ form at low octene concentrations, lower than 2 mol%. At higher octene concentration, the crystallization of the kinetically favored α form prevails.

## 1. Introduction

Copolymerization of propene with other α-olefins of different chain lengths to isotactic random copolymer is a well know strategy to modify the molecular structure and physical properties and mechanical behavior of isotactic polypropylene (iPP) and expand the possible applications of iPP [1,2,3]. The introduction of constitutional defects as comonomers of different sizes into polypropylene chains produces, generally, a decrease of melting temperature and density, resulting in higher clarity and an improvement of flexibility and ductility of iPP [1,4]. The efficient change of the mechanical properties depends on the size and concentration of the comonomer, and, in general, on the type of the incorporated defect. Therefore, understanding the effect of different defects on the crystallization and mechanical properties of iPP allows for a controlled modification of properties [4,5].

The effect of comonomers on the crystallization behavior of iPP and crystallization of the different polymorphic forms has been extensively investigated in samples prepared either with heterogeneous Ziegler–Natta catalysts [6,7,8,9,10,11,12,13,14,15,16,17,18,19,20,21,22,23,24,25,26,27,28,29,30,31,32,33] or homogeneous metallocene catalysts [34,35,36,37,38,39,40,41,42,43,44,45,46,47,48,49,50,51,52,53,54,55,56,57,58,59,60,61,62,63,64,65,66,67,68,69,70,71,72,73,74,75,76,77,78,79,80,81,82,83,84,85,86,87,88,89,90,91,92,93]. These studies have shown that modification of mechanical properties of iPP, as the deformation behavior, depends mostly on the crystallization of α and γ forms.

The α form is the stable form of iPP and generally crystallizes in the common conditions of crystallization from solution or from the melt and in fibers [5,94].

In standard highly stereoregular samples of iPP synthesized with heterogeneous Ziegler–Natta catalysts, the γ form has been observed only in low molecular mass samples [95], in copolymers of iPP with various comonomers [6], and by crystallization at high pressures [96,97,98,99,100].

The discovery of single-center homogeneous metallocene catalysts [101,102,103] allowed the synthesis of homogeneous samples of iPP that crystallize easily into the γ form in common conditions of crystallization at atmospheric pressure in samples of high molecular mass [104,105,106,107,108,109,110,111,112,113,114,115] and in random copolymers of iPP [34,35,36,37,38,39,40,41,42,43,44,45,46,47,48,49,50,51,52,53,54,55,56,57,58,59,60,61,62,63,64,65,66,67,68,69,70,71,72,73,74,75,76,77,78,79,80,81,82,83,84,85,86,87,88,89,90,91,92,93]. In fact, the γ form that crystallizes in iPP samples is characterized by chains containing defects of stereoregularity (as *rr* triads) or regioregularity (for instance 2,1 secondary propene units) [104,105,106,107,108,109,110,111,112,113,114,115], and constitutional defects, such as comonomers [34,35,36,37,38,39,40,41,42,43,44,45,46,47,48,49,50,51,52,53,54,55,56,57,58,59,60,61,62,63,64,65,66,67,68,69,70,71,72,73,74,75,76,77,78,79,80,81,82,83,84,85,86,87,88,89,90,91,92,93]. In these defective samples of iPP and in its copolymers with various comonomers, the γ form generally crystallizes in mixture with α form, and the relative amount of γ form increases with increasing the concentration of stereo-defects [104,105,106,107,108,109,110,111,112], regio-defects [115], and of comonomeric units [34,35,36,37,38,39,40,41,42,43,44,45,46,47,48,49,50,51,52,53,54,55,56,57,58,59,60,61,62,63,64,65,66,67,68,69,70,71,72,73,74,75,76,77,78,79,80,81,82,83,84,85,86,87,88,89,90,91,92,93]. In these conditions, and in the special case of copolymers, the crystallization of the γ form significantly modifies the mechanical behavior of iPP [1,4,75,78,86,87,88,89,90,91,92,116,117,118,119,120].

The easy crystallization of γ form in metallocene-based iPP and copolymers is related to the shortening of the length of the regular isotactic propene sequences due to the presence of defects randomly distributed along the chains generated by the catalyst. In fact, it is known that the crystallization of γ form is induced by short regular propene sequences, that is, when iPP chains contain any type of defect that interrupts the regular propene sequences [52,72,74,107,110]. The crystallization of α form is instead preferred when the regular propene sequences are very long, which are generated when the content of defects is low or when defects are segregated in blocks of the macromolecules [110,111,113,114].

In general, for iPP samples and iPP-based copolymers synthesized with heterogeneous multi-site Ziegler–Natta catalysts, the distribution of defects (and comonomeric units) along the macromolecules is not random, but they are segregated in blocks of the macromolecules [113,114,121,122]. Moreover, copolymer chains are characterized by mixtures of different macromolecules that may have different composition and type of distribution of the comonomers along the chains [123,124]. Therefore, in these systems, the regular propene sequences are generally much longer, giving crystallization of α form even for high concentration of defects. The nonrandom distribution of comonomers along the chains of Ziegler–Natta copolymers and the presence of other types of microstructural defects have so far prevented the study of the effect of a single comonomeric unit on the crystallization behavior and physical properties of iPP.

Copolymers of iPP and iPP homopolymer prepared with single-center metallocene catalysts are instead characterized by a more homogeneous molecular structure with a perfectly random and uniform distribution of molecular defects along the chain. Therefore, even a low content of defects decreases the length of the regular propene sequences, inducing the crystallization of the γ form [107,110,111,115].

The crystallization of α and γ forms depending on the different molecular structure and architecture has a great influence on the physical and mechanical properties of iPP [4,5,75,88,89,90,91,92,110,116,117,118,119]. Therefore, in general, the molecular architecture and topology of copolymers, from standard random to block or multiblock copolymers, greatly affects the crystallization behavior and properties of polymers because of the different length of crystallizable sequences [123,124,125,126,127,128,129,130,131,132,133].

The random distribution of defects and comonomers along the macromolecules allows considering iPPs and its copolymers synthesized with metallocene catalysts as model systems for the crystallization behavior of iPP, which is defined by the average length of the regular propene sequences that is directly related (inversely) to the concentration of defects [110]. Since the crystallization behavior of iPP depends on the length of regular propene sequences, the inclusion of defects in the crystals or their exclusion from the crystals plays a fundamental role. The inclusion of defects in the crystals gives longer crystallizable propene sequences, even for high concentrations of defects, whereas exclusion produces a shortening of the regular propene sequences [72,74,93,110]. This aspect is particularly relevant in the case of iPP-based copolymers with different α-olefins, as different comonomeric units may or may not be incorporated in the crystals of α or γ forms, depending on the size of comonomers and their compatibility with the crystal structures of the α and γ forms. In this context, detailed studies of the crystallization behavior and properties of copolymers of iPP with ethylene, butene, pentene, and hexene prepared with metallocene catalysts have been reported in the literature [34,35,36,37,38,39,40,41,42,43,44,45,46,47,48,49,50,51,52,53,54,55,56,57,58,59,60,61,62,63,64,65,66,67,68,69,70,71,72,73,74,75,76,77,78,79,80,81,82,83,84,85,86,87,88,89,90,91,92,93]. However, all these comonomers (ethylene, butene, pentene, and hexene) are in part included in the crystals and in part excluded from the crystals of α and γ forms, and their degree of inclusion and the partitioning of defects between the crystalline and amorphous phases have been also evaluated by solution and solid-state ^13^C NMR and ab initio calculations [51,61,108,109]. Therefore, only the effect of comonomers included in the crystals or only the effect of comonomers excluded from the crystals cannot be understood yet.

This paper reports an analysis of the crystallization behavior of isotactic propene-octene copolymers (iPPC8) synthesized with a metallocene catalyst. Octene units are completely excluded from the crystals of both α and γ forms, therefore, we aim at clarifying the specific effect of defects excluded from the crystals on the crystallization behavior of iPP. The crystallization behavior of iPPC8 copolymers is compared with those of copolymers of iPP with ethylene, butene, pentene, and hexene. The effect of the octene units excluded from the crystals on the crystallization of α and γ forms of iPP is analyzed and compared to those of the different mentioned comonomers that are partially included in the crystals of α and γ forms with a different degree of incorporation.

## 2. Materials and Methods

**Scheme 1:**
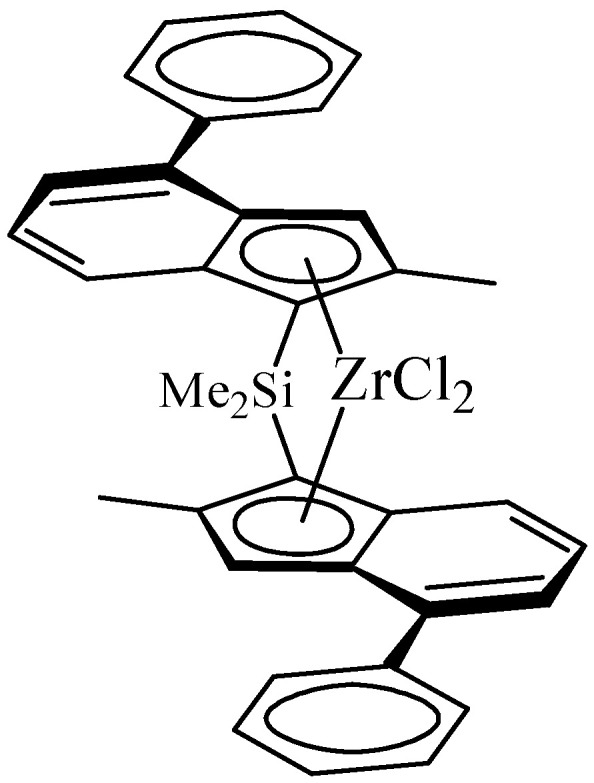
Structure of metallocene complex dimethylsilyl (2,2′-dimethyl-4,4′-diphenylindenyl)ZrCl_2_ used as catalyst for the synthesis of iPPC8 copolymers.

Propene–octene isotactic copolymers (iPPC8) were prepared with the metallocene catalyst of Scheme 1 [134] in combination with methylalumoxane (MAO) (from Lanxess, Cologne, Germany), as reported in ref. [79]. The used catalyst is highly isospecific in both homo- and copolymerizations, and the iPPC8 copolymers result in being highly isotactic and contain very low amounts of defects of stereoregularity and regioregularity, with only about 0.2 mol% of 2,1-eryhro propene units [134] (Table 1). Moreover, octene incorporation does not affect the molecular mass, and all samples present high values of molecular mass (Table 1).

The composition and comonomer distribution were determined by ^13^C NMR analysis [135,136] (Table 1). All spectra were obtained using a Bruker DPX-400 spectrometer operating in the Fourier transform mode at 120 °C at 100.61 MHz (Bruker Company, Billerica, Massachusetts, USA). The samples were dissolved with 8% wt/v concentration in 1,1,2,2-tetrachloroethane-d_2_ at 120 °C. The carbon spectra were acquired with a 90° pulse and 12 s of delay between pulses and CPD (WALTZ 16) to remove ^1^H-^13^C coupling. About 1500–3000 transients were stored in 32 K data points using a spectral window of 6000 Hz. For all copolymer samples, the peak of the propene methine carbon atoms was used as an internal reference at 28.83 ppm. The resonances were assigned according to ref. [135], and the 1-octene concentrations in the copolymers were evaluated from the constitutional diads PP, PO, and OO concentration (P = propene, O = octene). The NMR analysis showed that all the copolymers present a statistical distribution of comonomers (*r*_1_ × *r*_2_ ≈ 1) and homogeneous intermolecular composition.

The molecular masses and the dispersity were determined by gel permeation chromatography (GPC), using a Polymer Laboratories GPC220 apparatus equipped with a differential refractive index (RI) detector and a Viscotek 220R viscometer (Agilent Company, Santa Clara, CA, USA), on polymer solutions in 1,2,4-trichlorobenzene at 135 °C.

The calorimetry measurements were performed with differential scanning calorimeter (DSC) Mettler Toledo DSC-822 (Columbus, OH, USA) performing scans in a flowing N_2_ atmosphere and a scanning rate of 10 °C/min.

X-ray powder diffraction profiles were recorded with Ni filtered Cu Kα radiation by using an Empyrean diffractometer (Malvern Panalytical, Worcestershire, UK), performing continuous scans of the 2θ Bragg angle from 2θ = 5° to 2θ = 40°.

All samples of iPPC8 copolymers were isothermally crystallized from the melt at different crystallization temperatures (*T*_c_). Powder samples were melted at 200 °C and kept for 5 min at this temperature in a N_2_ atmosphere. They were then rapidly cooled to the crystallization temperature, *T*_c_, and kept at this temperature, still in a N_2_ atmosphere, for a time long enough to allow complete crystallization at *T*_c_. After the complete crystallization, the samples were quenched to room temperature and analyzed by X-ray diffraction and DSC. In the various isothermal crystallization experiments, the crystallization time is different depending on the crystallization temperature. The crystallization time necessary to have complete crystallization was evaluated by recording the crystallization exotherms in DSC and evaluating the crystallization kinetics. The crystallization time is about 2 h for low crystallization temperatures and about 2 weeks for the highest crystallization temperatures.

The degrees of crystallinity (*x*_c_) were determined from the powder diffraction profiles by the ratio between the crystalline diffraction area (*A*_c_) and the area of the whole diffraction profiles (*A*_t_ = *A*_c_ + *A*_am_), *x*_c_ = (*A*_c_/*A*_t_) × 100. The area of the crystalline phase (*A*_c_) has been determined subtracting a baseline and the scattering halo of the amorphous phase (*A*_am_) from the whole diffraction profile. For iPPC8 copolymer samples with low comonomer concentration, the amorphous halo has been obtained from the X-ray diffraction profile of a sample of atactic polypropylene. For iPPC8 copolymer samples with high octene concentration, the amorphous halo has been obtained from the X-ray diffraction profile of the amorphous sample iPPC8-6 with the highest octene concentration (15.9 mol%).

In samples that crystallize in mixtures of α and γ forms, the weight fraction of crystals of γ form *f*_γ_, with respect to that of the α form, was evaluated from the intensity of the (117)_γ_ reflection at 2θ = 20.1° of the γ form, with respect to that of the (130)_α_ reflection at 2θ = 18.6° of the α form, as the ratio: $f_{\gamma }=100\times (I{\left(117\right)}_{\gamma }/[I\left(117{)}_{\gamma }+I\left(130{)}_{\alpha }\right]\right)$. The intensities of (117)_γ_ and (130)_α_ reflections were measured from the area of the corresponding diffraction peaks above the diffuse amorphous halo in the X-ray powder diffraction profiles. The amorphous halo was obtained as described above. Next, it was scaled and subtracted to the X-ray diffraction profiles of the melt-crystallized samples. This method was applied to samples of iPPC8 copolymers of low octene concentrations that present X-ray diffraction profiles with well-defined and separated (117)_γ_ reflection at 2θ = 20.1° of the γ form and (130)_α_ reflection at 2θ = 18.6° of the α form. For samples with high octene concentrations that crystallize from the melt in disordered modifications of γ form intermediate between the α and γ forms [112], the structural disorder reduces the intensities of both (130)_α_ and (117)_γ_ reflections, and in limit of high degree of disorder in the γ crystals, both (130)_α_ and (117)_γ_ reflections disappear. In these cases, the absence of the (130)_α_ and (117)_γ_ reflections prevents the application of the method based on the intensities of the (130)_α_ and (117)_γ_ reflections, and the amount of γ form has been evaluated using the method described in ref. [112], based on the simulation of the diffraction profiles by calculating the X-ray diffraction as the sum of the contributions of the diffraction of crystals of α form and of the diffraction of disordered crystals of γ form. The amount of γ form in the mixture of α crystals and disordered crystals of γ form corresponds to that which gives the best agreement between experimental and calculated diffraction profiles.

## 3. Results and Discussion

**Figure 1:**
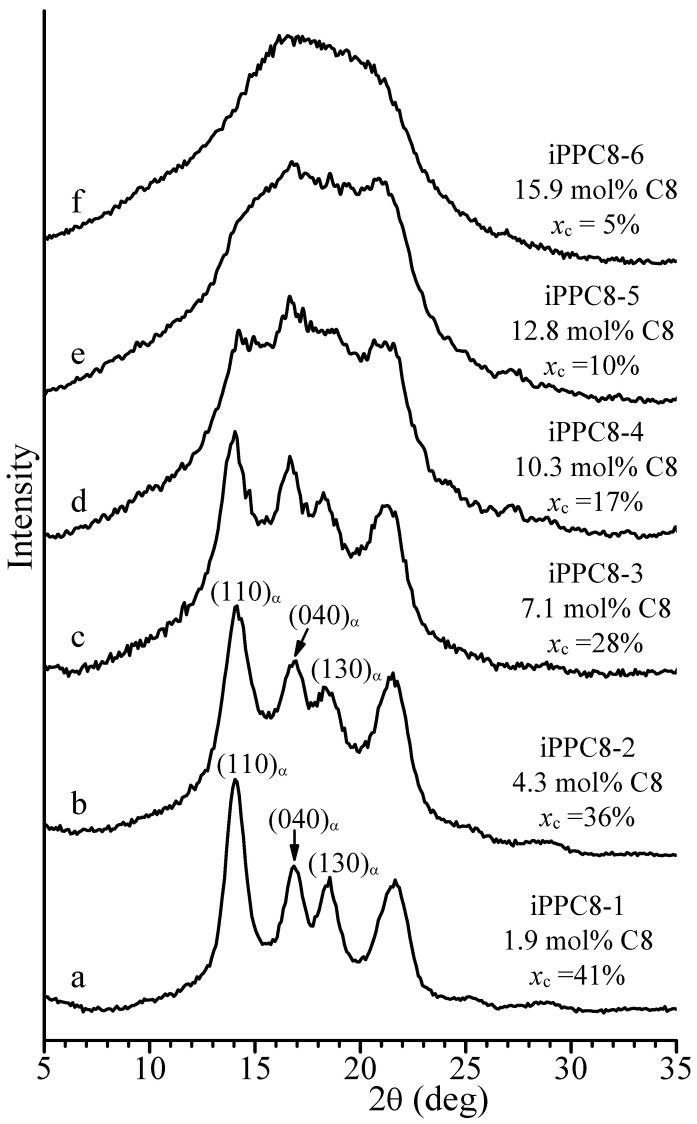
X-ray diffraction profiles of as-prepared samples of iPPC8 copolymers of the indicated octene concentration (**a**–**f**). The (110)_α_, (040)_α_, and (130)_α_ reflections at 2θ = 14°, 17°, and 18.6°, respectively, of the α form of iPP and the values of the degree of crystallinity *x*_c_ are indicated.

**Figure 2:**
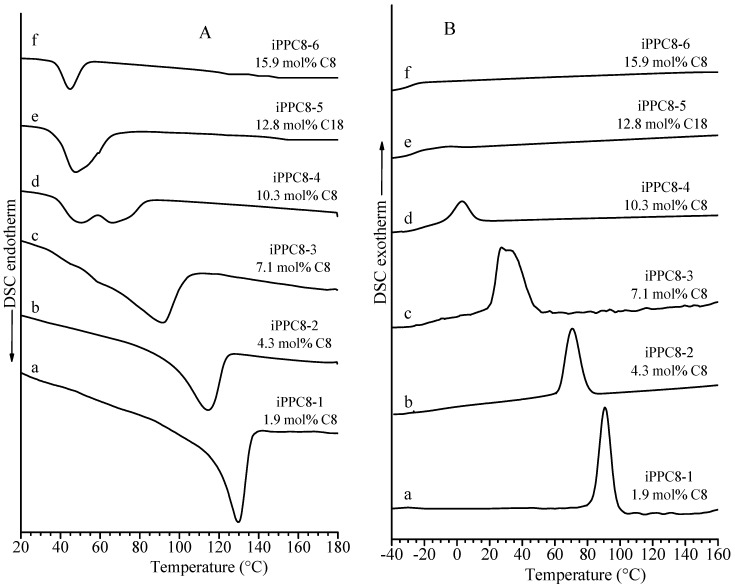
DSC heating (**A**) and cooling (**B**) curves of as-prepared samples of iPPC8 copolymers of the indicated 1-octene concentration (**a**–**f**).

The X-ray powder diffraction profiles of as-prepared (precipitated from the polymerization solution) samples of iPPC8 copolymers are shown in Figure 1. The values of the degree of crystallinity evaluated from the diffraction profiles are also shown in Figure 1. The corresponding DSC heating curves are reported in Figure 2A. These data indicate that the presence of octene produces a decrease of crystallinity and melting temperature with increasing octene concentration from *x*_c_ = 41% and *T*_m_ = 132 °C of the sample with 1.9 mol% of octene down to *x*_c_ = 5% and *T*_m_ = 45 °C of the sample with 15.9 mol% of octene. For octene concentrations higher than 16 mol%, the copolymers do not crystallize any more.

It is apparent that samples with octene content up to about 12 mol% crystallize in the α form, as indicated by the presence in the diffraction profiles a–d of Figure 1 of the (110)_α_, (040)_α_, and (130)_α_ reflections at 2θ = 14.1, 17.0, and 18.6°, respectively, of the α form of iPP, and the absence of the (117)_γ_ reflection at 2θ = 20.1° of the γ form. The diffraction profiles of samples with octene concentration higher than nearly 12 mol% present a diffuse halo with some shoulders or very weak and broad diffraction peaks (profiles e, f of Figure 1). This indicates that these samples are basically amorphous. In the sample iPPC8-5 with 12.8 mol% of octene (profile e of Figure 1), very small and broad reflections are indeed still observed at 2θ ≈ 14, 17, and 21°, corresponding to the (110)_α_, (040)_α_, and (111)_α_ reflections of the α form, and the amorphous scattering of the sample iPPC8-6 with 15.9 mol% of octene is clearly asymmetric with a broad peak at 2θ = 14° (profile f of Figure 1). This indicates that in the samples iPPC8-5 and iPPC8-6, a very small residual crystallinity (*x*_c_ = 10 and 5%, respectively), which can be attributed to disordered crystals of the α form, is still present [79]. This is also demonstrated by the DSC heating curves e and f of Figure 2A, which still present endothermic peaks at about 47 and 45 °C [79].

The DSC curves of the iPPC8 copolymers recorded during cooling from the melt are reported in Figure 2B. It is apparent that the crystallization temperature and enthalpy decrease with increasing octene concentration, and the samples iPPC8-5 and iPPC8-6 with 12 mol% and 15.9 mol% of octene do not crystallize by cooling from the melt. In the curves e and f of Figure 2B, only very small and broad exothermic signals are visible at very low temperature of nearly −10 °C. The DSC cooling curves recorded at low cooling rates of 2.5 °C/min (data not shown) are very similar to those of Figure 2B. The low crystallinity that these two samples show in the as-prepared specimens (profiles e and f of Figure 1 and Figure 2A) is due to further crystallization upon aging at room temperature.

These data indicate that the γ form of iPP does not crystallize in these iPPC8 copolymers, even at high octene concentrations. Instead, this occurs in copolymers of iPP with ethylene (iPPC2) [72], butene (iPPC4) [72,81,92], pentene (iPPC5) [82,90,91,93], and hexene (iPPC6) [68,69,74,77]. Moreover, Figure 1 also indicates that the trigonal δ form does not crystallize in these iPPC8 copolymers, as instead occurs in iPPC5 [73,82,90,91,93] and iPPC6 [68,69,70,74,77] copolymers.

The analysis of the 2θ positions of the (110)_α_ and (040)_α_ reflections of α form in the diffraction profiles of Figure 1 indicates that the Bragg distances of the diffracting planes are the same as those of the homopolymer, indicating that there is not expansion of the unit cell dimensions and that, therefore, octene co-units are excluded from the crystals of the α form. This behavior is different from those of iPPC4 [72,81,92], iPPC5 [73,82,90,91,93], and iPPC6 [68,69,70,74,75,77] copolymers, in which the comonomers are included, at least in part, in the crystals of α form with different degree of inclusion depending on the size and type of the comonomer unit. For iPPC6 and iPPC5 copolymers with high comonomer concentration, a huge amount of hexene and pentene comonomeric units is included in the unit cell of α form, inducing increase of crystal density that, in turn, induces crystallization of the δ form [68,69,70,73,74,75,77,82,90,91,93].

The exclusion of octene from the crystals of α and γ forms and the consequent absence of the crystallization of the trigonal δ form explains the fact that iPPC8 copolymers crystallize only up to 10–12 mol% of octene, whereas iPPC5 and iPPC6 copolymers crystallize up to very high pentene and hexene concentrations of about 55 mol% of pentene [73,82,90,91,93] and 25–30 mol% of hexene [68,69,70,74,75,77].

**Figure 3:**
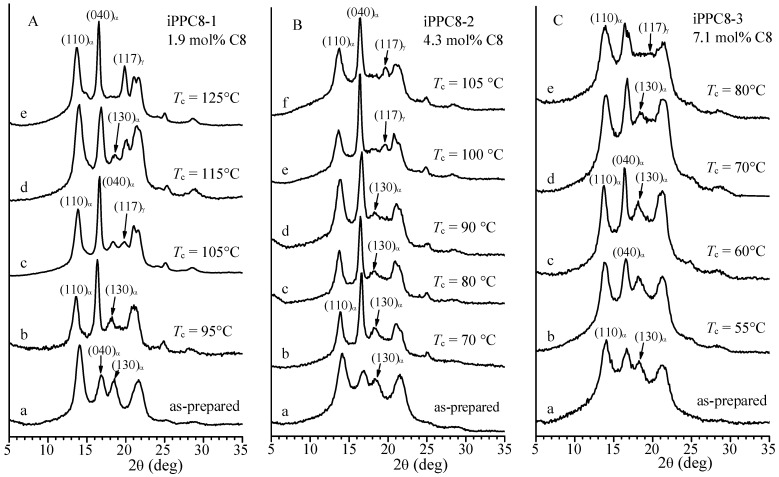
X-ray powder diffraction profiles of samples isothermally crystallized from the melt at the indicated crystallization temperatures *T*_c_ (**b**–**f**) of the samples iPPC8-1 with 1.9 mol% of octene (**A**), iPPC8-2 with 4.3 mol% of octene (**B**), and iPPC8-3 with 7.1 mol% of octene (**C**). The diffraction profiles of the as-prepared samples are also reported (profiles (**a**)). The (110)_α_, (040)_α_, and (130)_α_ reflections at 2θ = 14°, 17°, and 18.6°, respectively, of the α form of iPP and the (117)_γ_ reflection of the γ form at 2θ = 20.1°, are indicated.

To study the effect of the octene comonomeric units excluded from the crystals on the crystallization of α and γ forms, samples of iPPC8 copolymers have been isothermally crystallized from the melt at high crystallization temperatures in conditions close to the thermodynamic conditions. The diffraction profiles of samples of iPPC8 copolymers isothermally crystallized from the melt at different temperatures are reported in Figure 3. The diffraction profiles of the as-prepared samples, (already presented in Figure 1) are also reported in Figure 3 (profiles a) for comparison. The isothermal crystallizations have been performed only for the three samples of iPPC8 copolymers with low octene concentrations of 1.9, 4.3, and 7.1 mol% that crystallize from the melt and develop a significant degree of crystallinity. As discussed above, samples with higher octene concentration do not crystallize from the melt or develop very low crystallinity and crystallize by cold crystallization.

Samples of iPPC8 copolymers with low octene concentrations of 1.9 and 4.3 mol% crystallize at any crystallization temperature in mixtures of α and γ forms, as indicated by the presence of the (130)_α_ and (117)_γ_ reflections at 2θ ≈ 18.6° and 20.1° of the α and γ forms, respectively, in all the diffraction profiles of Figure 3A,B. In these two samples, the intensity of the (117)_γ_ reflection at 2θ = 20.1° of the γ form increases up to achieve a maximum, whereas the intensity of the (130)_α_ reflection at 2θ = 18.6° of the α form decreases with increasing crystallization temperature. This indicates that the relative amount of γ form increases, and that of the α form decreases, with increasing crystallization temperature up to achieve a maximum (Figure 3A,B).

The sample iPPC8-3 with higher octene concentration of 7.1 mol% instead crystallizes at all crystallization temperatures only in the α form, as indicated by the presence of only the (130)_α_ reflection at 2θ = 18.6° of the α form and the absence of the (117)_γ_ reflection at 2θ = 20.1° of the γ form in all the diffraction profiles of Figure 3C. Only at the highest crystallization temperature of 80 °C does a very small broad peak at about 2θ = 20.1° appear, while the (130)_α_ reflection at 2θ = 18.6° disappears (profile e of Figure 3C). This indicates development at high crystallization temperature of a small amount of γ form. However, the lack of well-defined (130)_α_ and (117)_γ_ reflections at 2θ = 18.6° and 20.1° of α and γ forms in the diffraction profile e of Figure 3C indicates that the sample iPPC8-3 crystallizes in a disordered modification of the γ form intermediate between the ordered α and γ forms [110,111,112]. Contrary to propene–pentene and propene–hexene copolymers that, for high comonomer concentration, crystallize in the trigonal δ form [74,93], no traces of the trigonal δ form are observed in all three samples of iPPC8 copolymers isothermally crystallized from the melt (Figure 3).

**Figure 4:**
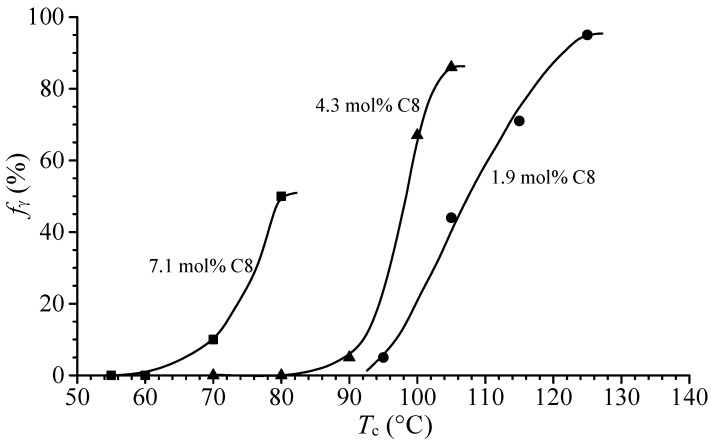
Relative amount of γ form (*f*_γ_) that crystallizes from the melt in samples of iPPC8 copolymers isothermally crystallized from the melt at the crystallization temperatures, *T*_c_, as a function of the crystallization temperature *T*_c_. (⬤) sample iPPC8-1 with 1.9 mol% of octene, (▲) sample iPPC8-2 with 4.3 mol% of octene, (■) sample iPPC8-3 with 7.1 mol% of octene.

**Figure 5:**
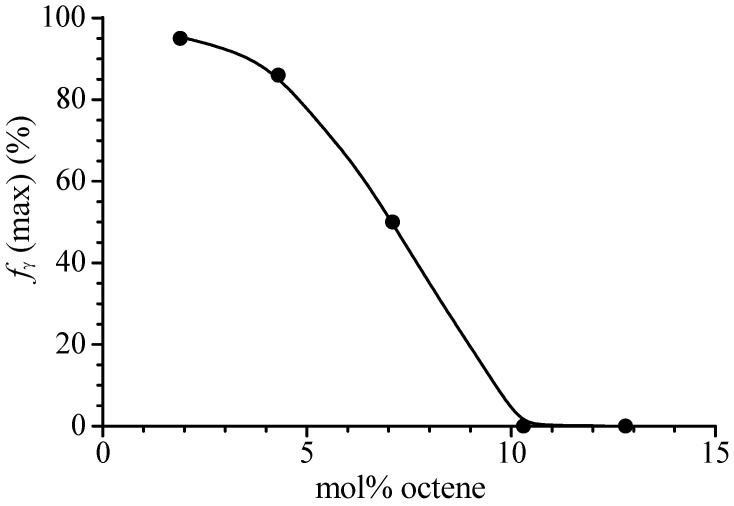
Maximum amount of γ form (*f*_γ_(max)) obtained in isothermal crystallization from the melt of iPPC8 copolymers (the highest values of Figure 4) as a function of octene concentration.

The values of the amount of γ form (*f*_γ_), with respect to the α form, that crystallizes in the isothermal crystallizations, determined from the intensities of (117)_γ_ and (130)_α_ reflections in the diffraction profiles of Figure 3, are reported in Figure 4 as a function of the crystallization temperature. For all samples, the amount of γ form increases with increasing crystallization temperature up to achieve a maximum *f*_γ_(max). In samples of different octene concentrations, a maximum amount of γ form is achieved at different crystallization temperatures (Figure 4). The values of the maximum amount of γ form (*f*_γ_(max)) that are obtained in each sample are reported in Figure 5 as a function of the octene concentration. It is apparent that the maximum amount of γ form achieves the highest value of 95% at the lowest octene concentration of 1.9 mol% and then decreases with increasing octene concentration down to a value of about 50% for the sample iPPC8-3 with 7.1 mol%. This sample always crystallizes in the α form and crystallizes in the disordered modification intermediate between the ordered α and γ forms only at the highest crystallization temperature (Figure 3C). For this sample (diffraction profile e of Figure 3C), because of the absence of both (130)_α_ and (117)_γ_ reflections at 2θ = 18.6° and 20.1° of α and γ forms, the amount of γ form has been calculated from the simulation of the diffraction profile by calculating the X-ray diffraction as the sum of the contributions of the diffraction of crystals of α form and of the diffraction of disordered crystals of γ form [112]. A value of *f*_γ_ = 50% has been obtained.

The behavior of iPPC8 copolymers of Figure 5 is completely different from the behaviors observed in iPPC2 [72], iPPC4 [72], iPPC5 [93], and iPPC6 [74] copolymers. In fact, the maximum amount of γ form achieved in each sample of different copolymers depends on the comonomer concentration and generally increases with increasing comonomer concentration, while in iPPC8 copolymers, the maximum amount of γ form decreases with increasing octene concentration (Figure 5).

**Figure 6:**
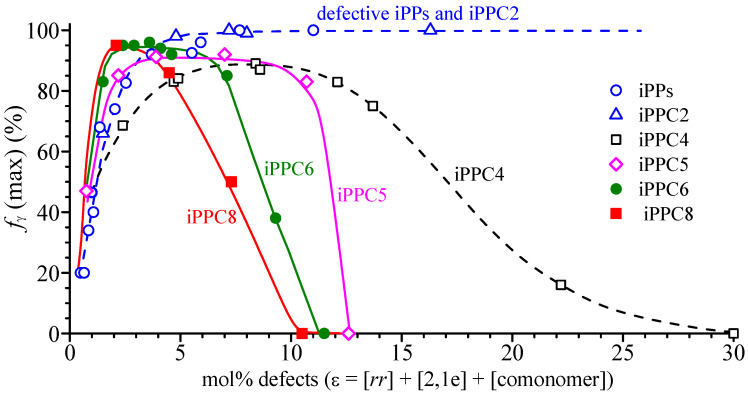
Maximum amount of γ form (*f*_γ_(max)) obtained in samples of iPPC8 copolymers isothermally crystallized from the melt (■) as a function of the total concentration of defects ε, compared to literature values found for stereoirregular iPPs (**○**) [110], and for copolymers iPPC2 (**△**) [72], iPPC4 (**☐**) [72], iPPC5 (**◇**) [93], and iPPC6 (●) [74].

The data of the maximum amount of γ form of iPPC8 copolymers of Figure 5 are also plotted in Figure 6 as a function of the total concentration of defects, defined as the sum of steric defects (*rr* diads), 2,1 regiodefects, and octene units, ε = [*rr*] + [1,2] + [octene], and compared with the values of *f*_γ_(max) observed and found in the literature for highly stereoregular iPP homopolymer [72,74,93,115], iPPC2 [72], iPPC4 [72], iPPC5 [93], and iPPC6 [74] copolymers. The literature values refer to copolymers synthesized with similar isospecific catalysts [72,74,93,115] that produce copolymers with isotacticity as high as that of the iPPC8 copolymers analyzed in this paper. Therefore, the differences among the different copolymers observed in Figure 6 are due to the different effects of the different comonomers on the crystallization of the α and γ forms. In the plot of Figure 6, the data of maximum amount of γ form produced by melt crystallizations of stereodefective iPPs containing different amounts of defects of stereoregularity (only *rr* diad defects) synthesized with various metallocene catalysts are also reported [110]. Figure 6 shows that, compared to the homopolymer sample of similar high isotacticity synthesized with the same or similar catalyst, for all copolymers the maximum amount of γ form rapidly increases with increasing comonomers concentration. For iPPC8 copolymers, the increase of *f*_γ_(max) from that of the homopolymer is very fast, and the highest value of *f*_γ_(max) is achieved at a very low octene concentration of 1.9 mol%. Then, for octene contents higher than 1.9 mol%, *f*_γ_(max) decreases (Figure 5 and Figure 6).

The compared data of Figure 6 indicate that for iPPC2 copolymers, *f*_γ_(max) increases with increasing ethylene content to achieve the maximum value of *f*_γ_(max) = 100%, corresponding to the crystallization of the pure γ form [72]. A similar effect is visible in the stereodefective samples of iPP homopolymer containing variable content of stereodefects (only *rr* diad defects) (Figure 6) [110]. In fact, for these iPP samples the values of *f*_γ_(max) increase with increasing concentration of *rr* defects and achieve the highest maximum value *f*_γ_(max) = 100% for *rr* defects content higher than 5–7 mol% (Figure 6). Both iPPC2 copolymers and defective iPPs crystallize from the melt in the pure γ form for ethylene and *rr* defect concentrations higher than 5–7 mol% [72,110]. This suggests that *rr* diad defects and ethene units provide a similar effect in inducing crystallization of γ form, and the values of *f*_γ_(max) of defective iPPs and iPPC2 copolymers in Figure 6 are interpolated by the same curve [72,110].

Different behaviors are instead observed in Figure 6 for the other copolymers. In fact, in copolymers iPPC8, iPPC6, iPPC5, and iPPC4, the maximum amount of γ form *f*_γ_(max) first increases, achieves the highest value of nearly 90–95% and then decreases with further increase of comonomer concentration (Figure 6). The rates of increase and then of decrease of *f*_γ_(max) with the comonomer concentration are, however, different in these copolymers. For low defects concentration (lower than 2–3 mol%), the increase of *f*_γ_(max) observed in iPPC8 copolymers is faster than that in iPPC6, iPPC5, and iPPC4 copolymers, whereas the increase of *f*_γ_(max) observed in iPPC6 copolymers is faster than that in iPPC5 and iPPC4 copolymers, and, finally, the increase of *f*_γ_(max) in iPPC5 copolymers is, in turn, faster than that in iPPC4 copolymers (Figure 6). The fastest increase of *f*_γ_(max) is, indeed, observed for iPPC8 copolymers characterized by the largest size comonomer. Moreover, the highest values of *f*_γ_(max) in iPPC5 and iPPC6 copolymers are obtained soon for low pentene and hexene concentrations (2–3 mol%), whereas in iPPC8 copolymers is achieved at the lowest comonomer concentration of 1.9 mol% (Figure 6). Furthermore, the highest value of the maximum amount of γ form in iPPC8 copolymers (95%) is higher than those in copolymers iPPC6, iPPC5, and iPPC4, whereas iPPC6 copolymers produce a maximum amount of γ form (90%) higher than those in copolymers iPPC5 and iPPC4. Finally, iPPC5 copolymers give a highest maximum amount of γ form higher than that developed in iPPC4 copolymers [72,74,93].

At high concentrations of comonomers, *f*_γ_(max) decreases in the three copolymers iPPC4, iPPC5, and iPPC6 down to *f*_γ_(max) = 0, corresponding to crystallization of the pure α form. The decrease of *f*_γ_(max) in iPPC6 copolymers is faster than in iPPC5 copolymers, which is, in turn, faster than in iPPC4 copolymers. In the case of iPPC8 copolymers, the decrease of *f*_γ_(max) from the highest value is faster than in iPPC6, iPPC5 and iPPC4 copolymers, because the value of *f*_γ_(max) = 50% is obtained already for the sample with 7.1 mol% of octene, which gives only a disordered modification of γ form with structure intermediate between those of the α and γ forms (Figure 3C). The drop off of *f*_γ_(max) starts at concentrations of comonomers of 2 mol% for iPPC8 copolymers, nearly 4–5 mol% in iPPC6 copolymers, 10–11 mol% in iPPC5 copolymers, and 14–15 mol% in iPPC4 copolymers (Figure 6) [72,74,93]. Moreover, for concentrations of pentene and hexene higher than 10–11 mol%, iPPC5 and iPPC6 copolymers do not crystallize any more in the γ form but crystallize in the α form or in mixtures of α and δ forms [74,93]. Instead, at these high concentrations of butene, iPPC4 copolymers continue to crystallize in a mixture of α and γ forms, and only for butene concentration higher than 30 mol% they crystallize in the pure α form [72]. In iPPC8 copolymers, crystallization of the γ form is no longer observed at octene concentrations higher than 7 mol%, and for this composition, iPPC8 copolymers do not crystallize or develop only very low crystallinity of the α form (Figure 5).

Since in the analyzed copolymers there is no effect of stereo- or regio-defects, the observed different behavior is due to the different effect of different comonomers on the crystallization of iPP, related to the inclusion of different comonomers into crystals of α and γ forms or their exclusion from the crystals. Defects as comonomers [53,72,74,90,91,92,93], as well as defects of stereoregularity [105,106,107,108,109,110,111] and regioregularity [108,115], give the same effect of interruption the propene sequences, shortening the average length of regular propene sequences <*L*_iPP_> and favoring the crystallization of the γ form. When the defects are incorporated in part or totally in the crystals of α and γ forms, the length of the crystallizable sequences increases, favoring the crystallization of the form that better accommodates the defect into crystals [72,74,90,91,92,93].

Ethylene, butene, pentene, hexene, and octene comonomers show different degrees of inclusion in crystals of iPP. A small amount of ethene is included in crystals of α and γ forms, and iPPC2 copolymers do not crystallize at high ethylene concentrations [51,52,72]. A high amount of butene is instead easily incorporated in the crystals of α form and, as a consequence, iPPC4 copolymers crystallize in the whole range of composition [61,72,92]. The degree of inclusion of pentene and hexene comonomeric units is very low in copolymers with low comonomer content but is very high in samples of high comonomer concentration [64,65,67,68,69,70,73,74,75,77,82,90,91,93]. Therefore, at low concentrations, pentene and hexene comonomeric units act as defects interrupting the regular propene sequences and inducing crystallization of the γ form [74,93]. At high comonomer concentrations, instead, the high fraction of co-units included in the crystals of the α form induces crystallization of the α form [68,69,73,74,77,93] and produces an increase of crystal density that then induces crystallization of the δ form for comonomer concentrations higher than about 15–16 mol% [68,69,70,73,74,77,82,90,91,93]. As discussed above, octene units are instead excluded from the crystals and are mainly segregated in the amorphous phase.

**Figure 7:**
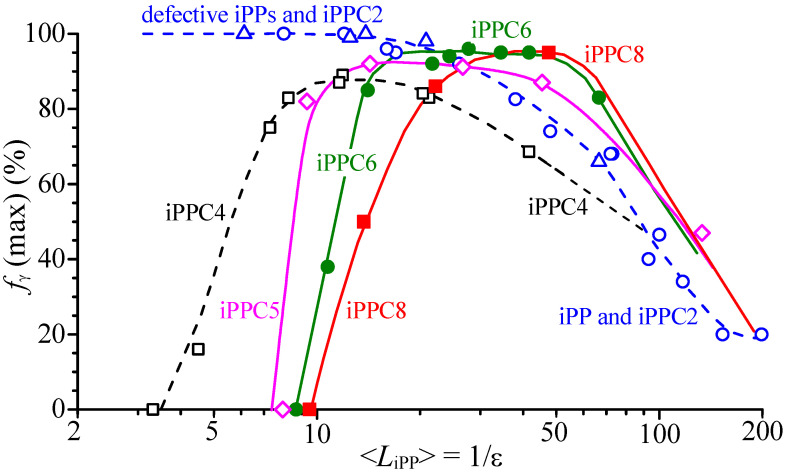
Maximum amount of γ form (*f*_γ_(max)) obtained in iPPC8 copolymers isothermally crystallized from the melt (■) as a function of the average length of regular propylene sequences <*L*_iPP_> compared to literature data of *f*_γ_(max) found for stereoirregular iPPs (**○**) [110], iPPC2 (**△**) [72], iPPC4 (**☐**) [72], iPPC5 (**◇**) [93], and iPPC6 (●) [74] copolymers.

The two competing effects of interruption of the regular propene sequences of defects excluded from the crystals and the inclusion of defects into crystals define the crystallization behavior of iPP. The interruption effect induces crystallization of γ form [72,74,93,110], whereas the inclusion effect induces crystallization of α and δ forms [68,69,70,72,73,77,82,90,91,92,93]. For random copolymers and, generally, for iPP chains characterized by random distribution of defects along the macromolecules, the average length of the regular propene sequences is inversely proportional to the total concentration of defects ε and can be evaluated as <*L*_iPP_> ≈ 1/ε [110]. The data of the maximum amount of γ form *f*_γ_(max) of Figure 6 of all copolymers and of defective iPPs [72,74,93,110] are reported in Figure 7 as a function of the average length of the regular propene sequences <*L*_iPP_>. For the different copolymers, different relationships between <*L*_iPP_> and *f*_γ_(max) have been obtained [72,74,93,110], and iPPC8 copolymers give a new different behavior.

The plot of Figure 7 indicates that the data of iPPC2 copolymers and of defective iPPs follow the same relation between *f*_γ_(max) and <*L*_iPP_>, which essentially corresponds to the interruption effect [72,110]. Ethylene co-units and *rr* stereodefects are mainly excluded from crystals (or partially included), and the effect of interruption and of shortening the length of the regular propene sequences prevails [72,110]. For the iPPC8 copolymers, octene defects are also mainly excluded from the crystals, and therefore, the interruption effect predominates and at octene concentrations lower than 2 mol% iPPC8 copolymers give the highest maximum amount of γ form, similar to that of stereodefective iPPs and iPPC2 copolymers (Figure 7). At higher octene concentration, the behavior of iPPC8 copolymers deviates from the master curve of iPPC2 and stereodefective iPPs, and the maximum amount of γ form does not depend anymore on the average length of regular propene sequences <*L*_iPP_> and decreases with increasing octene concentration and decreasing <*L*_iPP_>. This is due to the fact that the long octene units makes the crystallization of the γ form too much slow even for short values of <*L*_iPP_>, inducing crystallization of the kinetically favored α form, and prevents complete crystallization of both α and γ forms at octene concentrations higher than 12–13 mol%.

The three copolymers iPPC4, iPPC5, and iPPC6 give three different relationships between *f*_γ_(max) and <*L*_iPP_> (Figure 7), because different amounts of the three comonomers are included in the crystals of the α form [72,74,93]. At low concentrations of comonomer (lower than 5–6 mol% and <*L*_iPP_> of 200-30 monomeric units), the included amounts of pentene and hexene are low, as in the case of ethylene, and the effect of interruption prevails inducing crystallization of the γ form. The bigger the comonomer, the more efficient the interruption effect, and the higher the amount of γ form. In fact, for this composition, iPPC5 and iPPC6 copolymers give an amount of γ form higher than those of iPPC2 copolymers and defective iPPs but lower than that of iPPC8 copolymers (Figure 7) [74,93]. High amounts of butene co-units are, instead, included in the crystals of α form, even at low concentrations, and iPPC4 copolymers give a maximum amount of γ form lower than those of iPPC5, iPPC6, and iPPC8 copolymers, and lower than those of iPPC2 copolymers and streoirregular iPPs (Figure 7) [72].

For comonomer concentrations higher than 5 mol% and <*L*_iPP_> lower that 20–30 monomeric units, hexene, pentene, and butene units are incorporated in the α form to very high extents with corresponding increase of crystalline density, and the inclusion effect with the stabilization of the α form prevails over the interruption effect, inducing the crystallization of the α and δ forms, with a fast decrease of the maximum amount of γ form down to zero for iPPC6, iPPC5, and iPPC4 copolymers (Figure 7) [72,74,93]. A fast or slow decrease of *f*_γ_(max) depends on the fast or slow increase of crystal density, which depends on the size of the included comonomer. Therefore, the decrease of *f*_γ_(max) is faster in iPPC6 copolymers because of the bigger hexene units, and the decrease of *f*_γ_(max) in iPPC5 copolymers is faster than that of iPPC4 copolymers. It is worth noting that the fastest decrease of the maximum amount of γ form in iPPC8 copolymers is not due to incorporation of octene units in the crystals of α form, but, as mentioned above, is rather correlated to the fact that the excluded octene units make the crystallization of γ form too slow, inducing the faster crystallization of the kinetically favored α form, and then, at high concentrations, completely prevents crystallization of both α and γ forms (Figure 7).

These data on iPPC8 copolymers and the comparison with the literature demonstrate that the crystallization behavior of iPP may be described in terms of a model that defines a double role exerted by defects, the interruption of the regular propene sequences, and the inclusion effect. The crystallization of α, γ, and δ forms of iPP depends on which effect prevails, which in turn depends on the size and type of the defect.

## 4. Conclusions

Random isotactic propene–octene copolymers with octene concentrations ranging from 1.9 to 16 mol% have been synthesized with a homogeneous single center metallocene catalyst. The as-polymerized samples with octene content up to 10–12 mol% crystallize in the α form of iPP, whereas for higher octene concentration, the samples are basically amorphous or show very low crystallinity. The melting temperatures decrease with increasing octene content from 132 °C of the sample with 1.9 mol% of octene down to 47–45 °C for the samples with 12.8 and 15.9 mol% of octene.

Three samples of iPPC8 copolymers with low octene concentration of 1.9, 4.3, and 7.1 mol% have been isothermally crystallized from the melt at different crystallization temperatures. The samples crystallize at any crystallization temperature in mixtures of α and γ forms and the relative amount of γ form increases with increasing crystallization temperature and achieves a maximum value, which depends on the octene concentration. Contrary to propene–pentene and propene–hexene copolymers that, for high concentrations of pentene and hexene, crystallize in the δ form [74,93], the crystallization of the trigonal δ form has not been observed in all three samples of iPPC8 copolymers crystallized from the melt.

The behavior of iPPC8 copolymers is completely different from the behaviors observed in iPPC2 [72], iPPC4 [72], iPPC5 [93], and iPPC6 [74] copolymers. In fact, the maximum amount of γ form achieved in each sample of different copolymers depends on the concentration of comonomer and generally increases with increasing comonomer content, while in iPPC8 copolymers, the maximum amount of γ form decreases with increasing octene concentration. This different behavior is due to the fact that in iPPC8 copolymers octene units are excluded from the crystals, giving only the interruption effect that shortens the length of the regular propene sequences, inducing crystallization of the γ form. A maximum amount of γ form is achieved at low octene concentrations of nearly 1.9 mol%. At higher octene concentration, the amount of γ form crystallized from the melt rapidly decreases.

In copolymers of iPP with butene, pentene, and hexene, the comonomer units are, instead, incorporated in the crystals of α form to a very high extent. At a high concentration of comonomers, the inclusion effect that favors crystallization of the α form prevails over the interruption effect. The efficiency of the incorporation effect depends on the size of comonomers, as a rapid or slow increase of density is obviously correlated to the size of the incorporated comonomer.

For iPPC8 copolymers, the observed fastest decrease of the maximum amount of γ form and the consequent crystallization of the α form for high octene concentrations is not due to incorporation of octene units in the crystals of α form, but is due to the fact that the excluded octene units make the crystallization of γ form too slow, inducing the faster crystallization of the kinetically favored α form and, then, at high concentration, prevent the crystallization of both α and γ forms.

The reported results on crystallization of iPPC8 copolymers and the comparison with the literature demonstrate that the crystallization behavior of iPP may be described in terms of a model that defines two effects exerted by defects: the interruption of the regular propene sequences and the inclusion effect. The crystallization of α, γ, and δ forms of iPP depends on which effect prevails, which in turn depends on the size and type of the defect.

## Figures and Tables

**Table 1:** Feed (mL octene) and copolymer composition (mol% octene), melting temperature of as-prepared samples (*T*_m_), molecular mass (*M*_w_), and dispersity (*M*_w_/*M*_n_) of iPPC8 copolymers [79].

Sample	Feed (mL Octene)	Composition (mol% Octene)	*T*_m_ (°C) ^a^	*M*_w_^b^	*M*_w_ */M*_n_^b^
iPPC8-1	1	1.9	132.5	398,576	2.1
iPPC8-2	4	4.3	114.4	257,274	2.0
iPPC8-3	6	7.1	91.8	302,205	2.1
iPPC8-4	7	10.3	72.6/53.2	252,380	2.1
iPPC8-5	8	12.8	47.3	230,985	2.0
iPPC8-6	10	15.9	44.9	225,750	2.0

^a^ Determined from DSC heating curves recorded at 10 °C/min. ^b^ Determined by GPC.

## Data Availability

The data in this study are available on reasonable request from the corresponding author.
